# Enhancing Surgical Outcomes: Evaluating the Impact of Implementing the World Health Organization Surgical Safety Checklist—A Prospective Cross-Sectional Study

**DOI:** 10.1055/s-0044-1800917

**Published:** 2024-12-12

**Authors:** ElMuhtadi B. Y. Gasoma, Mohamed A. Marouf

**Affiliations:** 1 T&O Surgery Department, Swansea Bay UHB, United Kingdom; 2 Cardiothoracic Surgery Department, Royal Sultanate Hospital, Muscat, Oman

**Keywords:** surgical safety checklist, surgical complications, sign in, time out, sign out

## Abstract

**Background** Globally, surgical care plays a vital role in health care. Unfortunately, complications arise in approximately 5 to 18% of surgical procedures. However, research has shown that following the surgical safety checklist provided by the World Health Organization (WHO) can significantly reduce these complications and surgery-related fatalities. The objective of this study was to assess the accuracy and completion of the WHO Surgical Safety Checklist.

**Materials and Method** From October 3, 2022 to February 28, 2023, a comprehensive observational study was conducted involving 300 patients who underwent elective and emergency surgeries. The completion rates for the different stages of surgery, namely sign-in, time-out, and sign-out, were determined using the SPSS 22.0 software.

**Result** In our research, we specifically examined the completion of the checklist for 300 patients who underwent important procedures within a span of 5 months. For each patient, we ensured that their identities, procedures, and consent were verified with a 100% success rate. Additionally, we thoroughly checked the anesthesia equipment and drugs for safety in every case. Furthermore, all essential images were consistently displayed for each patient, achieving a 100% adherence to this crucial aspect of the process.

**Conclusion** In general, the level of completeness of the checklist was satisfactory across the sign-in, time-out, and sign-out phases. While this study did not assess the specific outcomes, it is presumed that incomplete data may have exposed patients to potential perioperative complications.

Surgical procedures are a vital part of health care around the world, with approximately 234 million operations performed every year. 1 2 This number is higher than the number of births each year. 3 However, surgery also carries a risk of complications, which happen in 5 to 18% of cases. 4 5 These complications can lead to serious harm or death for patients, affecting 1 out of every 310 operations. 6 7 8 Therefore, it is important to prevent medical errors as much as possible. 7 One way to do this is to use the World Health Organization (WHO) Surgical Safety Checklist, which is a global tool to improve patient safety during surgery. 9 The checklist covers the essential aspects of surgical care, such as infection prevention, anesthesia safety, teamwork and communication, and quality measurement. 10 11 There is strong evidence that nearly half of the complications or adverse outcomes resulting from surgical operations can be avoided. 11 12 Studies have shown that using the checklist can reduce the rate of major complications from 11 to 7% and reduce the rate of mortality by 53% (from 1.5 to 0.8%). 2 7 The checklist has four main components that correspond to different stages of the surgical process. 13 The use of the checklist (following all four components) has increased over time and has been associated with lower rates of postoperative problems and mortality. 14 15 However, the effectiveness of the checklist depends on how well it is implemented in each hospital. Some challenges include integrating it into the workflow and measuring its impact on safety. 16 To ensure successful implementation, hospital leaders and staff need to be actively involved in the process, communicate and collaborate across disciplines, provide training and feedback, and conduct regular audits. 2 14 15 17 18 It has been shown that there is a direct link between better outcomes and the use and adherence to the checklist. 19 Therefore, to achieve the full benefits of the checklist, it is necessary to ensure proper compliance and implementation. The purpose of this study is to evaluate how well the WHO checklist is completed at Ribat University Hospital operating room (OR).

## Aims and Objectives

### Aim

The aim of this study was to assess the completion of all the surgery checks and briefing/debriefing for each operative and surgical procedure at Ribat University Hospital.

### Objectives

The main objectives of this study are: (1) to identify the missing steps in completing the surgical safety checklist; (2) to ensure that the right patient and the right site are operated on by the OR team; (3) to enhance the safety of anesthesia practices; and (4) to facilitate good communication among the OR team.

## Materials and Methods

A prospective observational cross-sectional study was conducted at Ribat University Hospital from September 03, 2022 to February 28, 2023.

### Audit Population

All surgical procedures done at Ribat University Hospital from September 03, 2022 to February 28, 2023.

### Audit Sample

All surgical procedures done during the study period.

### Data Collection Method and Analysis

The information was collected by direct observation and a checklist-based chart review. The audit proforma was created by converting the standards into question forms with the integrated checking options. Google Forms were used to enter the data, which were then exported into SPSS version 22.0 for analysis. Descriptive analysis using a three-level indication of compliance using the scoring method described in Box 1 was performed. Results are expressed in frequencies and percentages using table and figure. The work has been reported in line with the STROCSS criteria. 20

**Table:** Box 1 Details of the scoring method

The auditor utilized the subsequent scoring methodology when reviewing the record: 0 = If there is no sign that information was attempted to be documented. 1 = If insufficient information is documented. 2 = If some of the crucial details are documented. 3 = if the majority of the data are documented. 4 = if every detail of the information is documented. n/a = if the criterion is not relevant.
This meant that, if 150 patients were audited on each cycle, each standard may result in a score of 600. For instance, if they mark surgical site on all 150 patients, the compliance rate would be 100% and the overall score would be 600. If 120 patients have got their surgical site marked, the overall score would be 480, giving compliance of 80%. When n/a was inputted for a standard that did not apply to any of the patients, the spreadsheet automatically deducted 4 points from the projected result. Prior to converting the scores into compliance rates for the hospital, the scores for all procedures were obtained. The three-level indication system assures that standards with an overall compliance of 80% or higher are high, those with an overall compliance of 50 to 79% are moderate, and those with an overall compliance of less than 50% are low.

### Audit Standards

The WHO has created a worldwide surgical safety checklist to reduce the frequency of surgical complications by strengthening the commitment of clinical professionals to address safety issues in surgical settings. The checklist is divided into three sections: before induction of anesthesia, before skin incision, and before any team member exits the OR. The gold standard was a 100% completion rate of high-quality, safe surgery checks before each operation or surgical procedure ( Table 1 ).

**Table 1:** Standards of surgical safety drafted by World Health Organization

	Standards	Target	Evidence	Data source	Exception
Part I:	Before induction of anesthesia				
1	Confirm patients' identity, procedure and consent	100%	WHO guideline	Direct observation/interview	
2	Mark surgical site	100%	»	»	
3	Check anesthesia machine and medications	100%	»	»	
4	Known allergy	100%	»	»	
5	Difficult airway/aspiration	100%	»	»	
6	Risk of bleeding >500 mL	100%	»	»	7 mL/kg in children
Part II:	Before start of surgical incision				
7	All team members introduce themselves by name and role	100%	»	»	
8	Surgeon, anesthetist, and registered practitioner confirm verbally patient name, planned procedure, site, and position	100%	»	»	
9	Critical/unanticipated steps the surgeon may announce to the team	100%	»	»	
10	Patient-specific concern for anesthetist	100%	»	»	
11	Nurses' confirmation about the sterility of instrumentation	100%	»	»	
12	Antibiotic prophylaxis within the last 60 minutes	100%	»	»	
13	Essential imaging displayed	100%	»	»	
PART III:	Before any member of the team leaves the operating room				
14	Nurse verbally confirms name of procedure	100%	»	»	
15	Confirm instruments, swabs, and sharps counts are complete	100%	»	»	
16	Specimens labeled by patient name	100%	»	»	No specimen
17	Any equipment problem needs to addressed	100%	»	»	If all equipments functional
18	Report key concerns for the recovery room professionals	100%	»	»	

## Result

**Fig. 1:**
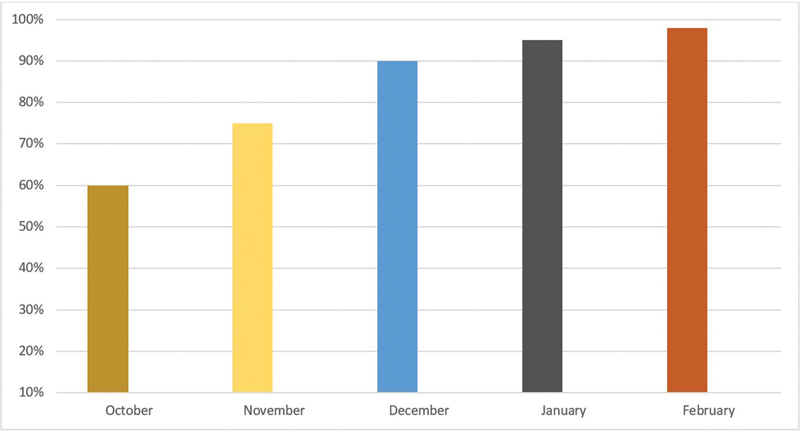
Checklist completions by month during implementation.

We examined the completion of the checklist for 300 patients who underwent major operations in 6 months in our study. The identity, procedure, and consent of all patients (100%) were verified as the standard requires in its first part (before anesthesia was administered). The anesthesia machine and medications were also scrutinized for safety for all patients. The allergic status of the patient was queried and documented in 97.01% of the patients. Essential images were displayed for all patients (100%; Table 2 ). Results for all standards were categorized into three indicators and coded using the system outlined in Box 1 for all cycles. Table 3 displays the percentage of standards that fell into each level when the results were compiled for the Ribat University Hospital. This audit summary shows that compliance seems to be continuously improving. There are now fewer moderate standards and more high standards. The highest completeness of the checklist was seen in February and the lowest completeness of the checklist was seen in October ( Fig. 1 ).

**Table 2:** Completed and missed items in the checklists over the 6 months analyzed

	Standards	Completed	%	Missed	%
Part I:	**Sign in**				
1	Confirm patients' identity, procedure, and consent	300	100	0	0
2	Mark surgical site	294	98.04	6	1,96
3	Check anesthesia machine and medications	300	100	0	0
4	Known allergy	291	97.01	9	2,99
5	Difficult airway/aspiration	291	97.04	9	2,96
6	Risk of bleeding >500 mL	289	96.5	11	3,5
Part II:	**Time out**				
7	All team members introduce themselves by name and role	271	90.4	29	9,6
8	Surgeon, anesthetist, and registered practitioner confirm verbally patient name, planned procedure, site, and position	294	98.2	6	1.8
9	Critical/unanticipated steps the surgeon may announce to the team	282	94.03	18	5.97
10	Patient-specific concern for anesthetist	286	95.4	14	4.6
11	Nurses' confirmation about the sterility of instrumentation	290	96.7	10	3.3
12	Antibiotic prophylaxis within the last 60 minute	281	93.8	19	6.2
13	Essential imaging displayed	300	100	0	0
Part III:	**Sign out**				
14	Nurse verbally confirms name of procedure	271	90.5	29	9.5
15	Confirm instruments, swabs, and sharps counts are complete	287	95.8	13	4.2
16	Specimens labeled by patient name	294	98.2	6	1.8
17	Any equipment problem needs to addressed	283	94.5	17	5.5
18	Report key concerns for the recovery room professionals	279	93.1	21	6.9

**Table 3:** Standards in each three-level indication

Indicator	Percentage of standards for October	Percentage of standards for November	Percentage of standards for December	Percentage of standards for January	Percentage of standards for February
**High** (80–100% compliance)	50% (9)	60% (11)	70% (13)	90% (16)	95% (17)
**Moderate** (50–79% compliance)	35% (6)	30% (5)	30% (5)	10% (2)	5% (1)
**Low** (<50% compliance)	15% (3)	10% (2)	0% (0)	0% (0)	0% (0)

## Discussion

**Fig. 2:**
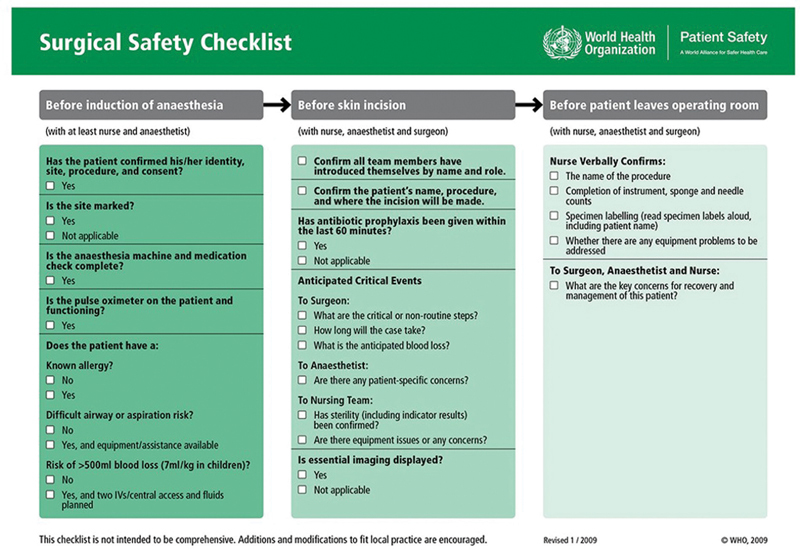
World Health Organization Surgical Safety Checklist.

The WHO checklist has three main parts, which are done at specific times during surgery: the first part (Sign-in) is before giving anesthesia to the patient; the second part (Time-out) is before making the surgical incision; and the third part (Sign-out) is before transferring the patient to the recovery room ( Fig. 2 ). Important information can be reviewed, shared, and communicated between all team members involved in surgery at each of these times. The checklist is meant to improve surgical outcomes and, as a result, health care quality in general. However, its introduction and sustainability are always a big challenge. The Sign-in period was more completed than the Time-out and Sign-out periods in this study. These items are important in avoiding the most common mistakes that seriously harm patients. 11 This finding is almost similar to a study done in India. 5 In addition, confirming patients' identity, procedure, and consent was 100% completed. This finding is nearly the same as a study done at the University of Gondar in Ethiopia, where only 1.9% was missed in their study. 6 In the current study, the anesthesia machine and medications check were completed in all patients. This finding is almost consistent with a study done at Yekatit 12 referral Hospital, Addis Ababa, Ethiopia. 21 However, even though they are essential steps, items of aspiration risk, anticipation of a difficult airway, and estimated blood loss were not checked in some cases; all of them could cause death. 22 One of the WHO Surgical Safety Checklist's main goals is to encourage communication among the surgical team. 23 In the Time-out period, surgical teams are supposed to mention their name and role to each other. In this period, some surgical team members did not mention their name and role to the others. However, this finding is higher than a study done in Thailand, in which the majority of the surgical team failed to introduce their name and functional role to others. 23 Essential imaging was displayed during surgery in all patients. This finding is almost in line with studies conducted in different institutions. 5 6 23 24 In this study finding, the Sign-out period was less performed compared with other sections. However, this finding is higher than most study findings conducted in different study areas. 6 10 24 25 26 27 The presence of adequate OR nurse staff, which reduces the workload of nurses needed to prepare for subsequent procedures, may be the cause of this disparity.

## Conclusion

While checklists have the potential to enhance surgical safety, their effectiveness depends on how well they are implemented. The overall completeness of the checklist in the sign-in, time-out, and sign-out periods was satisfactory. Furthermore, health care professionals have recognized the usefulness of using the checklist frequently during emergency cases. To promote more regular usage and enhance communication, additional training and focus on actual checklist utilization are recommended.

## Recommendations

Enhancing the WHO Surgical Safety Checklist can lead to improved patient outcomes, increased team communication, and a safer surgical environment. Here are some recommendations to consider:

- Incorporate digital solutions:
  - Electronic checklist: implement an electronic version of the checklist that can be accessed via tablets or computers. This can allow real-time updates and storage of completed checklists.
  - Integration with electronic health records (EHRs): link the checklist with EHR systems to automatically pull patient information and surgical details, reducing manual data entry errors.
- Customization for specialized procedures:
  - Procedure-specific modules: create modules or sections tailored to specific surgical procedures (e.g., orthopedic, cardiac, neurosurgery) to address unique risks and considerations.
  - Anesthesia checklist: develop a separate checklist or module focused on anesthesia-related safety measures and considerations.
- Enhanced communication:
  - Team introductions: add a step for team introductions at the beginning of the checklist to improve communication and team cohesion.
  - Time-outs: include mandatory “time-outs” before incision and before the patient leaves the OR to confirm critical information and ensure everyone is on the same page.
- Patient engagement:
  - Patient verification: incorporate a step where the patient verifies their identity, the procedure to be performed, and the surgical site to ensure correct patient and procedure matching.
  - Informed consent: ensure that informed consent has been obtained and documented for the procedure.
- Postoperative care:
  - Recovery checklist: develop a checklist for postoperative care to ensure a smooth transition to recovery, including pain management, monitoring of vital signs, and patient education.
  - Follow-up plan: create a plan for postoperative follow-up and communicate it to the patient and their caregivers.
- Training and education:
  - Regular training: provide regular training sessions for surgical teams on the use of the checklist and its importance in enhancing patient safety.
  - Feedback mechanism: establish a feedback mechanism where team members can provide input on the checklist's effectiveness and suggest improvements.
- Multidisciplinary collaboration:
  - Include nursing and anesthesia teams: involve nursing and anesthesia teams in the development and implementation of the checklist to ensure a multidisciplinary approach.
  - Feedback from multiple specialties: seek feedback from surgeons, nurses, anesthesiologists, and other health care professionals from various specialties to make the checklist more comprehensive and applicable.
- Data collection and analysis:
  - Performance metrics: define performance metrics to evaluate the effectiveness of the checklist in improving surgical outcomes and reducing complications.
  - Continuous improvement: analyze collected data regularly to identify areas for improvement and update the checklist accordingly.
- Cultural and organizational adoption:
  - Leadership support: gain support from hospital leadership and engage them in promoting the adoption and implementation of the enhanced checklist.
  - Cultural integration: integrate the checklist into the organizational culture by emphasizing its importance in daily surgical practice and celebrating its successes.
- Translation and accessibility:
  - Multiple languages: ensure the checklist is available in multiple languages to cater to a diverse patient population and health care workforce.
  - Accessible formats: provide the checklist in accessible formats (e.g., large print, braille) for patients with disabilities and health care professionals with specific needs.

Implementing these recommendations can help enhance the WHO Surgical Safety Checklist's effectiveness, ensuring safer surgical practices, improved team communication, and better patient outcomes. Regular review and updates based on feedback and new evidence will further support continuous improvement in surgical safety.
